# Macrophage Inflammatory Proteins (MIPs) Contribute to Malignant Potential of Colorectal Polyps and Modulate Likelihood of Cancerization Associated with Standard Risk Factors

**DOI:** 10.3390/ijms25031383

**Published:** 2024-01-23

**Authors:** Jarosław Wierzbicki, Iwona Bednarz-Misa, Łukasz Lewandowski, Artur Lipiński, Anna Kłopot, Katarzyna Neubauer, Małgorzata Krzystek-Korpacka

**Affiliations:** 1 Department of Minimally Invasive Surgery and Proctology, Wroclaw Medical University, 50-556 Wroclaw, Poland; jaroslaw.wierzbicki@umw.edu.pl; 2 Department of Biochemistry and Immunochemistry, Wroclaw Medical University, 50-368 Wroclaw, Poland; iwona.bednarz-misa@umw.edu.pl (I.B.-M.); lukasz.lewandowski@umw.edu.pl (Ł.L.); anna.klopot@umw.edu.pl (A.K.); 3 Department of Clinical Pathology, Wroclaw Medical University, 50-556 Wroclaw, Poland; artur.lipinski@umw.edu.pl; 4 Department of Gastroenterology and Hepatology, Wroclaw Medical University, 50-556 Wroclaw, Poland; katarzyna.neubauer@umw.edu.pl

**Keywords:** CCL3, CCL4, CXCL2, CCL19, malignancy risk, colorectal cancer, adenoma, adenocarcinoma, chemoprevention, dysplasia

## Abstract

Better understanding of molecular changes leading to neoplastic transformation is prerequisite to optimize risk assessment and chemopreventive and surveillance strategies. Data on macrophage inflammatory proteins (MIPs) in colorectal carcinogenesis are scanty and their clinical relevance remains unknown. Therefore, transcript and protein expression of CCL3, CCL4, CXCL2, and CCL19 were determined in 173 and 62 patients, respectively, using RT-qPCR and immunohistochemistry with reference to polyps’ characteristics. The likelihood of malignancy was modeled using probit regression. With the increasing malignancy potential of hyperplastic–tubular–tubulo-villous–villous polyps, the expression of *CCL3*, *CCL4*, and *CCL19* in lesions decreased. *CCL19* expression decreased also in normal mucosa while that of *CXCL2* increased. Likewise, lesion *CCL3* and lesion and normal mucosa *CCL19* decreased and normal *CXCL2* increased along the hyperplasia–low–high dysplasia grade. The bigger the lesion, the lower *CCL3* and higher *CXCL2* in normal mucosa. Singular polyps had higher *CCL3*, *CCL4*, and *CCL19* levels in normal mucosa. *CCL3*, *CCL4* and *CXCL2* modulated the likelihood of malignancy associated with traditional risk factors. There was no correlation between the protein and mRNA expression of CCL3 and CCL19. In summary, the polyp-adjacent mucosa contributes to gaining potential for malignancy by polyps. MIPs may help in specifying cancerization probability estimated based on standard risk factors.

## 1. Introduction

Despite recent progress in early diagnostics and new treatment modalities, colorectal cancer (CRC) remains the third in incidence and the second in prevalence and mortality among cancers worldwide, with a discreet sex-related variation [1]. The American Cancer Society has recently publicized data on CRC in the USA, estimating the probability of its lifetime occurrence to be 4.3% in males, this being higher only in prostate and lung cancers, and 3.9% in females, being higher only in breast and lung cancers. These three cancer types are prognosed to account for 48% of new cancer diagnoses in males and 52% in females. Data gathered over the last half-century indicate a steady decline in CRC incidence and mortality in USA, with estimated rates, respectively, at 1.5% and 2% per year since 2012 [2]. However, as 90% of new cases and 95% of deaths occur in individuals of 50 years of age and older [3], the overall downward trend managed to mask the disturbing 2% per year increase in CRC cases as well as 1.2% increase in mortality per year in people under 50 years of age [2]. Worldwide, in turn, CRC is on the rise, with currently the highest incidence and mortality rates in Western Europe. It is projected to result in 3.2 million diagnoses and 1.6 million deaths in 2040 as compared to 1.9 million diagnoses and 930,000 deaths in 2020. The increasing trends are particularly noticeable in younger adults [4].

The phenomenon of downward trends in incidence and mortality can be attributed to successful endoscopic surveillance implemented in high-risk individuals and the partially successful screening programs based on various fecal tests with subsequent endoscopy if justified. They allow for the diagnosis of cancer at earlier stages and thereby increase the possibility of its successful resection and reduce CRC-associated mortality. Moreover, screening programs enable the early detection of precancerous lesions and their timely removal contributing, in turn, to lowering CRC incidence [2,5,6].

It is believed that the incidence reduction in and the reversal of upward trends for younger adults can be achieved by chemoprevention. The known biology of colorectal cancer with well-defined precancerous stage and the time it takes for benign lesions to become malignant give us a unique opportunity for intervention aimed at reversing, preventing, or at least delaying the transformation [6]. Only a small percentage of polyps become malignant and the propensity of adenomas to become cancerous is higher the more numerous, larger, and villous they are and the higher their dysplasia grade [7]. Still, these characteristics do not explain all the risk. Therefore, novel targets for chemoprevention as well as candidates for malignancy risk predictors are sought after in order to improve and/or optimize screening methods and tactics, surveillance schedules, and chemopreventive strategies [8]. To achieve this, a detailed understanding of molecular background of neoplastic and malignant transformation is a necessity. Just as benign lesions rather than cancer cells are a better model for studying the malignant process, the analysis of changes occurring in polyp-adjacent non-transformed tissue is likely to contribute to greater understanding of the mechanisms causing the transformation of normal cells [9].

As Hanahan and Weinberg aptly pointed out [10], inflammation enables cancer. Accordingly, patients with chronic inflammatory bowel diseases (IBDs) are more likely to develop CRC. Compared to sporadic CRC, however, the molecular pathway leading to colitis-associated cancer is distinct. The inflammation-caused dysplasia is more difficult to detect and, consequently, patients’ prognosis is worse and mortality higher [11,12]. While inflammation is a driving force of mutagenesis and thus neoplastic transformation in IBD-associated cancer [11,12], it is a critical factor for tumor progression in sporadic CRC [13,14]. Inflammation involvement in earlier, precancerous phases is less clear, although increasingly appreciated [15] since infiltrates of immune cells in adenomas have been demonstrated [16]. Moreover, the extent of infiltration is proportional to adenoma size and dysplasia grade, implying that inflammatory and immune responses play an active role in carcinogenesis [16]. Therefore, targeting their initiators and propagators is the most intuitive and the best evaluated strategy of CRC chemoprevention. Non-steroidal anti-inflammatory drugs (NSAIDs), especially aspirin, are effective in preventing adenoma recurrence and have been shown to reduce the size and number of adenomas, both in experimental settings and human studies [12,17,18,19,20,21,22].

Infiltrates of immune cells in precancerous lesions [16] strongly suggest the involvement of chemokines. Yet, data on their presence and relevance in precancerous stages are surprisingly scanty, especially regarding those less popular ones. Macrophage inflammatory proteins (MIPs) belong to C-C (CCL3/MIP-1α, CCL4/MIP-1β, CCL19/MIP-3β) and C-X-C (CXCL2/MIP-2α/GROb) chemokine families. They recruit a number of immune cells, including monocytes, neutrophils, dendritic cells (DCs), T lymphocytes, NK cells, and myeloid-derived suppressor cells (MDSCs). MIPs are released by epithelial cells, including neoplastic cells, fibroblasts, macrophages, neutrophils, MDSCs, and mesenchymal stem cells (MSCs) (review [23,24]). Only by virtue of the cells they recruit, MIPs can play a double role in carcinogenesis depending on the context. They can either facilitate it, by promoting the proliferation, survival, and migration of cancer cells as well as angiogenesis, metastasis, and escape from immunosurveillance, or impede it by attracting anti-cancer proinflammatory subsets of immune cells. We have previously explored MIPs’ association with CRC and demonstrated the ability of novel oxicam analogues to modulate MIPs’ expression and secretion by colorectal adenocarcinoma cell lines [25]. Herein, we pursued MIPs’ relationship with precancerous polyps by determining their expression patterns in transformed and non-transformed tissue, at the transcript and protein level, with reference to known malignancy risk factors to assess their relevance for risk stratification and chemotherapy.

## 2. Results

The relative mRNA (qPCR) and protein (IHC) expression of CCL3, CCL4, CXCL2, and CCL19 in paired tissue samples—polyp-to-normal (P/N)—obtained from patients with polyps was determined and related to the risk factors of malignant transformation.

### 2.1. Expression Patterns of Genes Coding for Selected MIP Chemokines with Respect to Polyps’ Characteristics

#### 2.1.1. MIPs’ Expression in Neoplasms

*CCL3*, *CCL4*, *CXCL2*, and *CCL19* expression was analyzed as normalized relative quantities (NRQ) of chemokine transcripts in patient-matched samples of lesions and morphologically normal mucosa and expressed as expression fold-change. Except for *CXCL2*, upregulated in polyps, remaining chemokines were downregulated in polyps as compared to patient-matched normal mucosa. The downregulation of *CCL4* was non-significant (Table 1).

The individual analysis of polyps with adenocarcinomas (AC) and precancerous lesions of classic adenoma (A) and alternative serrated (H) transformation pathways showed opposite trends in serrated (upregulation) and classic (downregulation) pathways, significant for *CCL3* and non-significant for *CCL4* (Table 1).

Hyperplastic polyps had a significantly higher expression of *CCL3* than adenomas: 1.97 NRQ (95% *CI*: 0.64–6.08) vs. 0.7 NRQ (0.57–0.84), *p* = 0.015. Both hyperplastic polyps and adenomas had significantly higher *CCL19* expression than polyps with carcinomas: 1.84 (0.49–6.9) and 0.53 (0.38–0.74) vs. 0.03 (0–0.88), *p* = 0.002. The expression of *CCL4* and *CXCL2* in polyps did not differ significantly. There were no significant differences in MIPs’ expression in normal mucosa.

#### 2.1.2. Association of MIPs’ Expression with Lesion’s Histological Type

Except for *CXCL2*, which was upregulated in lesions as compared to normal mucosa regardless of polyp type, other genes presented changes in opposite direction in hyperplastic polyps (an upregulation in lesions) and polyps from traditional adenoma-carcinoma sequences (a downregulation in lesions). Still, only the fold-change in *CCL3* expression differed significantly. With hyperplastic polyps (H) and carcinomas in the polyp (AC) excluded, none of the downward trends in chemokine expression from tubular (T) through tubulo-villous (T-V) to villous (V) adenomas were statistically significant (Table 2). 

**Figure 1:**
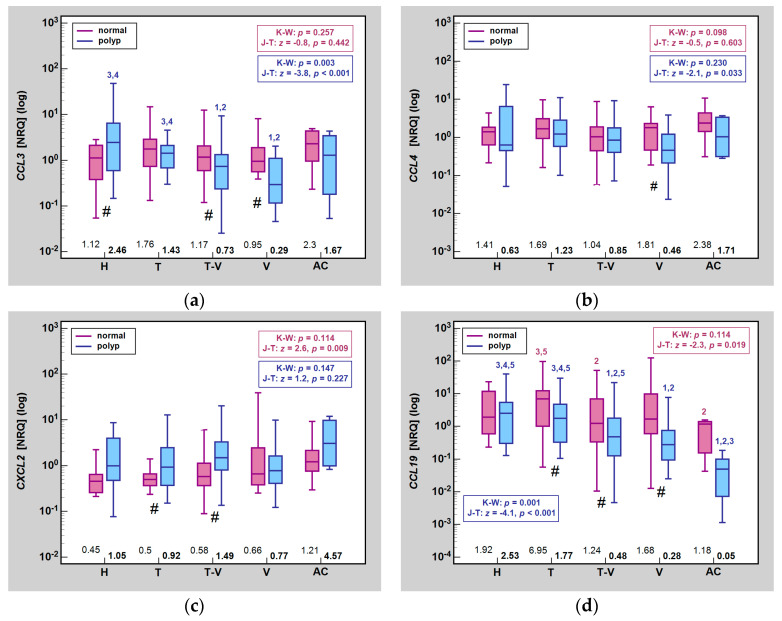
Impact of histological type on MIP expression in normal mucosa and polyps: (**a**) *CCL2*; (**b**) *CCL4*; (**c**) *CXCL2*; (**d**) *CCL19*. Data presented as medians (bars inside boxes) with *IQR* (boxes) and 99% *CI* (whiskers). In addition, median values are stated above X axis (bolded for gene expression in polyps). Significant differences between histological types are indicated by blue (polyp tissue) and purple (normal mucosa) numbers above whiskers. ^1^, significantly different from H; ^2^, significantly different from T; ^3^, significantly different from T-V; ^4^, significantly different from V; ^5^, significantly different from AC; #, significant difference between polyp and normal mucosa (independent analysis with Mann-Whitney *U* test). H, hyperplastic polyps; T, tubular adenoma; TV, tubulo-villous adenoma; V, villous adenoma; AC, adenocarcinoma; NRQ, normalized relative quantities; K-W, Kruskal-Wallis *H* test; J-T, Jonckheere-Terpstra test; *IQR*, interquartile range; *CI*, confidence interval.

A fold-change does not indicate whether the observed effect resulted from alterations in the expression pattern in the lesion, adjacent normal mucosa or both. Figure 1 presents an effect of the histological type on chemokine expression, indicating significant differences between histological types within the same tissue type (polyps and, separately, normal mucosa) as well as the differences between median chemokine expression in the lesion vs. normal mucosa in patients stratified by polyp histological type.

For *CCL3*, the histological type significantly affected its expression in polyp tissue, being higher in H and T than in T-V and V lesions, but not in normal mucosa. The Jonckheere-Terpstra test showed a significant downward trend in *CCL3* expression in the lesions, reflecting their increasing propensity for malignant transformation. A change in *CCL3* expression between the lesion and normal mucosa was significant in H, T-V, and V polyps (Figure 1).

For *CCL4* and *CXCL2*, there were no significant differences in gene expression either in lesions or normal tissue between polyps of different histological types. However, Jonckheere-Terpstra test showed a significant downward trend for *CCL4* in polyps and an upward trend for *CXCL2* in normal mucosa. In patients stratified based on polyp type, *CCL4* expression differed significantly between lesion and normal mucosa in V while *CXCL2* expression differed between lesion and normal mucosa in T and T-V (Figure 1).

For *CCL19*, changes were observed in both polyps and normal mucosa. In lesions, gene expression was the highest in H, significantly higher as compared to T-V, V, and AC. It was the lowest in AC, significantly lower also than in T and T-V. Significant differences between histological types could also be observed for T and T-V as well as T and V. There was a significant downward trend in *CCL19* expression, both in lesions and in normal mucosa. As for the latter, gene expression was the highest in T and it was significantly higher than in T-V and AC. Of analyzed histological types, significant differences in *CCL19* expression between lesion and normal tissue were observed for T, T-V, and V while the same albeit not statistically significant tendency (*p* = 0.053) was found in AC (Figure 1).

#### 2.1.3. Association of MIP Expression with Dysplasia Grade

The fold-change in chemokine expression did not differ significantly between low- and high-grade dysplasia. There was, however, significant difference between hyperplasia and dysplasia in the case of fold-change in *CCL3* expression (Table 3).

**Figure 2:**
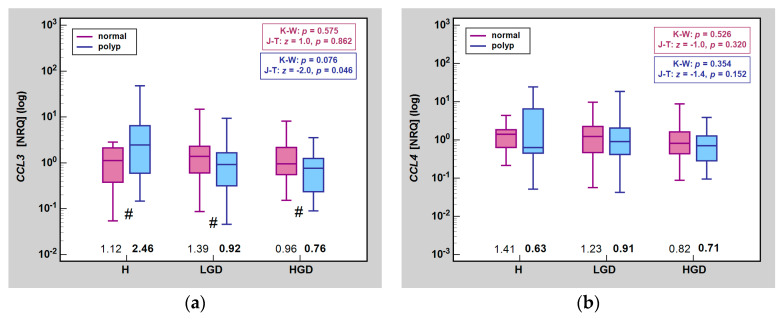

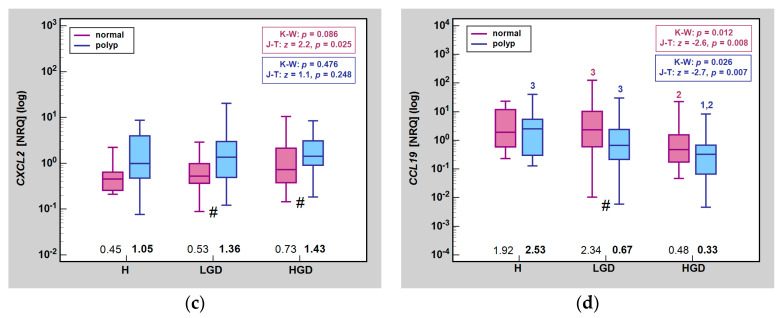
Impact of dysplasia grade on MIP expression in normal mucosa and polyps: (**a**) *CCL2*; (**b**) *CCL4*; (**c**) *CXCL2*; (**d**) *CCL19*. Data were analyzed using Mann–Whitney *U* test and presented as medians (bars inside boxes) with *IQR* (boxes) and 99%*CI* (whiskers). In addition, median values are stated above X axis (bolded for gene expression in polyps). ^1^, significantly different from H; ^2^, significantly different from LGD; ^3^, significantly different from HGD; #, significant difference between polyp and normal mucosa (independent analysis with Mann–Whitney *U* test). H, hyperplasia; LGD, low-grade dysplasia; HGD, high-grade dysplasia; NRQ, normalized relative quantities; K-W, Kruskal–Wallis *H* test; J-T, Jonckheere-Terpstra test; *IQR*, interquartile range; *CI*, confidence interval.

When analyzing a sequence from no dysplasia (hyperplasia) through low-grade to high-grade dysplasia, we observed a downward trend in *CCL3* expression in lesions and *CCL19* expression in lesions and normal mucosa as well as an upward trend in *CXCL2* expression in normal mucosa. *CCL19* was the only chemokine significantly lower in high-grade than low-grade dysplasia, both in lesions and normal mucosa (Figure 2).

#### 2.1.4. Association of MIP Expression with Lesion Size

Polyps were stratified based on their size into three categories < 10 mm (small), 10–19 mm (medium), and ≥20 mm (large). 

None of the calculated fold-changes in chemokine expression differed significantly for polyps of different size. Only fold-change in *CXCL2* tended to be higher in medium polyps (Table 4).

**Figure 3:**
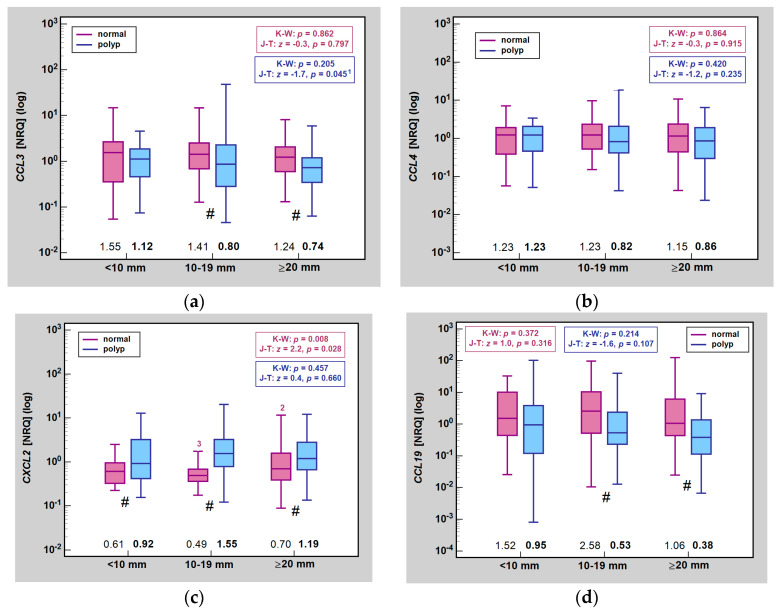
Impact of lesion size on MIP expression in normal mucosa and polyps: (**a**) *CCL2*; (**b**) *CCL4*; (**c**) *CXCL2*; (**d**) *CCL19*. Data presented as medians (bars inside boxes) with *IQR* (boxes) and 99% *CI* (whiskers). In addition, median values are stated above X axis (bolded for gene expression in polyps). Significant differences between size categories are indicated by blue (polyp tissue) and purple (normal mucosa) numbers above whiskers. ^1^, one-sided probability; ^2^, significantly different from 10–19 category; ^3^, significantly different from ≥20 mm category; #, significant difference between polyp and normal mucosa (independent analysis with Mann–Whitney *U* test; NRQ, normalized relative quantities; K-W, Kruskal–Wallis *H* test; J-T, Jonckheere-Terpstra test; *IQR*, interquartile range; *CI*, confidence interval.

Unpaired analysis on a larger set of samples confirmed lack of significant differences in *CCL4* and *CCL19* expression with respect to polyp size, both in lesions and normal mucosa. There were no significant differences in *CCL3* expression between different polyp size categories, both in lesions and normal mucosa. In lesions, however, there was a significant downward trend in expression from the highest in small to the lowest in large polyps. In turn, polyp size significantly affected *CXCL2* expression in normal mucosa, which displayed an upward trend along with increasing polyp size and a significant difference between medium and large polyps. Chemokine expression in lesions did not differ with respect to polyp size (Figure 3).

#### 2.1.5. Association of MIP Expression with Number/Character of Polyps

None of the fold-changes in chemokine expression differed significantly with respect to number/character of polyps (Table 5).

**Figure 4:**
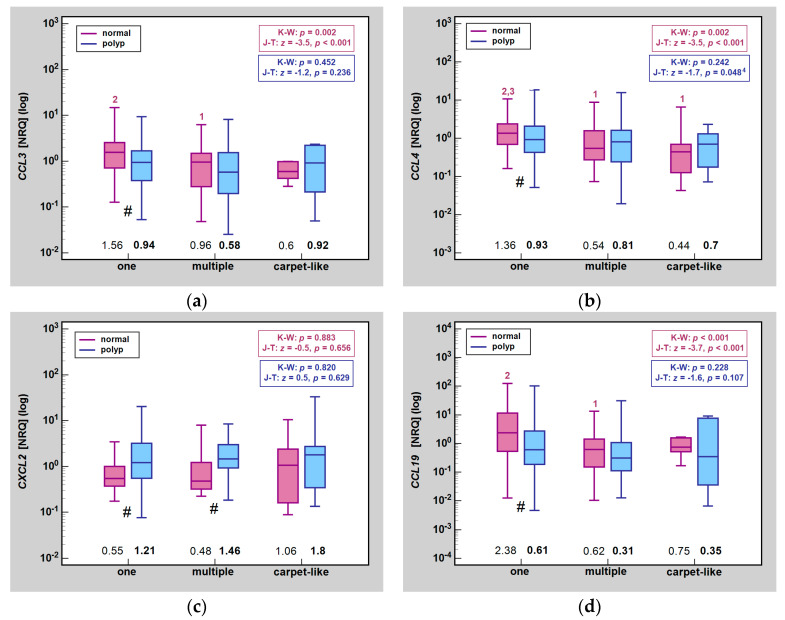
Impact of number/character of polyps on MIP expression in normal mucosa and polyps: (**a**) *CCL2*; (**b**) *CCL4*; (**c**) *CXCL2*; (**d**) *CCL19*. Data presented as medians (bars inside boxes) with *IQR* (boxes) and 99% *CI* (whiskers). In addition, median values are stated above X axis (bolded for gene expression in polyps). Significant differences between size categories are indicated by blue (polyp tissue) and purple (normal mucosa) numbers above whiskers. ^1^, significantly different from one; ^2^, significantly different from multiple; ^3^, significantly different from carpet-like; ^4^, one-sided probability; #, significant difference between polyp and normal mucosa (independent analysis with Mann–Whitney *U* test; NRQ, normalized relative quantities; K-W, Kruskal–Wallis *H* test; J-T, Jonckheere-Terpstra test; *IQR*, interquartile range; *CI*, confidence interval.

In unpaired analysis, *CCL3* expression was significantly lower in singular than multiple polyps in normal mucosa but did not differ significantly in lesions. Likewise, *CCL4* expression in normal mucosa was significantly higher in singular polyp as compared to multiple polyps and extensive carpet-like lesions. There were no significant differences in lesions, although a downward trend could be observed. The expression of *CXCL2*, either in normal mucosa or lesion, was unaffected by number or character of polyps. *CCL19* expression in lesions was unaffected as well but displayed a significant downward trend in normal mucosa and was significantly lower in multiple polyps as compared to singular polyp (Figure 4).

#### 2.1.6. Association of MIP Expression with Polyp Sublocation

Fold-change expression for *CCL3* and *CCL4* between polyp and normal tissue indicated significantly upregulated relative expression in lesions in right-sided polyps, contrary to other sublocations, where chemokines in lesions were relatively downregulated. Since similar chemokine expression levels could be observed between left-sided and rectal polyps, the results for these sublocations were pooled together and compared to right-sided polyps. Such analysis showed significant differences. Fold-change in *CXCL2* expression in lesions in the right-sided colon was insignificantly more upregulated and that of *CCL19* was insignificantly less downregulated as compared to other sublocations (Table 6).

**Figure 5:**
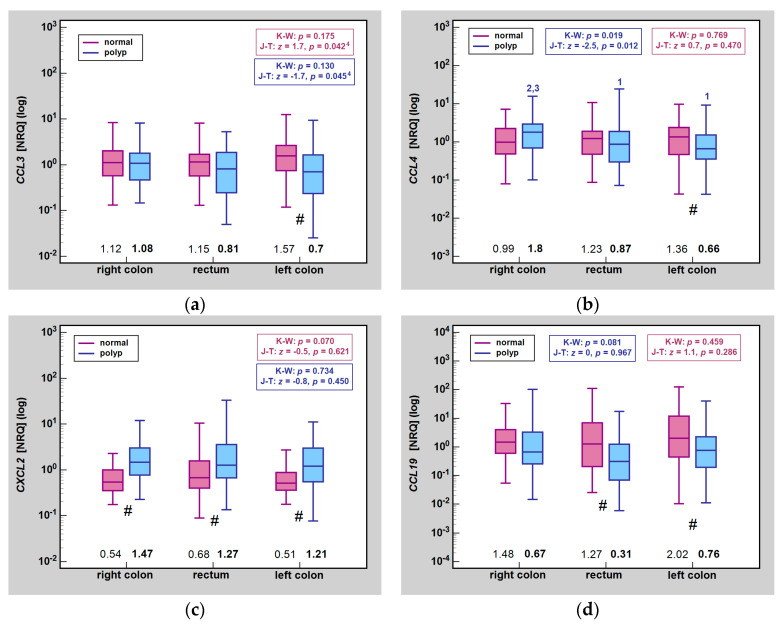
Impact of lesion sublocation on MIP expression in normal mucosa and polyps: (**a**) *CCL2*; (**b**) *CCL4*; (**c**) *CXCL2*; (**d**) *CCL19*. Data presented as medians (bars inside boxes) with *IQR* (boxes) and 99% *CI* (whiskers). In addition, median values are stated above X axis (bolded for gene expression in polyps). Significant differences between size categories are indicated by blue (polyp tissue) and purple (normal mucosa) numbers above whiskers. ^1^, significantly different from right colon; ^2^, significantly different from rectum; ^3^, significantly different from left colon; ^4^, one-sided probability; #, significant difference between polyp and normal mucosa (independent analysis with Mann–Whitney *U* test; NRQ, normalized relative quantities; K-W, Kruskal–Wallis *H* test; J-T, Jonckheere–Terpstra test; *IQR*, interquartile range; *CI*, confidence interval.

There were no significant between-group differences related to lesion sublocation in the expression of *CCL3*, *CXCL2*, and *CCL19*, both in normal mucosa and in polyps. However, *CCL3* expression displayed a significant downward trend with lesion sublocations ordered as follows: right colon–rectum–left colon, as well as an upward trend in normal mucosa (Figure 5). Pooled analysis of rectal and left-sided polyps showed median expression in lesions to be significantly lower than in right-sided ones (*p* = 0.043). In normal mucosa, in turn, *CCL3* expression tended to be significantly higher in left sided colon than right-sided and rectum (*p* = 0.063).

*CCL4* expression in lesions located in the right colon was significantly higher than in lesions located in the rectum or left colon while that in normal mucosa did not differ (Figure 5).

### 2.2. Modeling of Malignant Transformation Probability Based on Change in MIP Expression

Ordinal probit regression was applied to calculate the cumulative probability of changes in chemokine expression in lesions and normal mucosa and their interplay to describe propensity of individual polyps for malignancy. The likelihood of malignant transformation was assessed in terms of known risk factors associated with polyp characteristics, such as histological type, dysplasia grade, and size. Estimated polynomial probit models are summarized in terms of probit function (*ϕ*) and corresponding *p*-value for Wald’s z′ statistics in Tables S1–S3.

#### 2.2.1. Histological Type

As indicated by the significant but small negative *ϕ*, an upregulation of *CCL4* expression in the polyp would lower the likelihood of malignancy associated with polyp’s histological type. A downregulation of *CCL4* in polyp would increase the likelihood of malignancy. However, concomitant increase in *CCL4* expression in normal mucosa would reverse the trend (positive *ϕ* for “normal × polyp” interaction); therefore, malignancy likelihood would increase with *CCL4* upregulation in both the lesion and normal mucosa (Table 7).

*CXCL2* upregulation in normal mucosa is likely to decrease the likelihood of transformation resulting from histological type. The effects of *CCL3* and *CCL19*, although statistically significant, are negligible (Table 7).

#### 2.2.2. Dysplasia Grade

An increase in the polyp-to-normal ratio (P/N) of *CCL3* expression would rise the probability of malignant transformation in addition to dysplasia grade. The effect, as indicated by respective *ϕ*, is moderate. The negative function for an interplay between gene expression in tissues and P/N specifies a significant but too-weak effect to alter this trend (Table 8).

#### 2.2.3. Polyp Size

An increase in *CXCL2* expression in normal mucosa is likely to diminish the probability of malignancy associated with polyp size, as indicated by the relatively high, negative value of *ϕ* (Table 9). 

### 2.3. Expression of MIP Proteins in Colorectal Neoplasms

**Figure 6:**
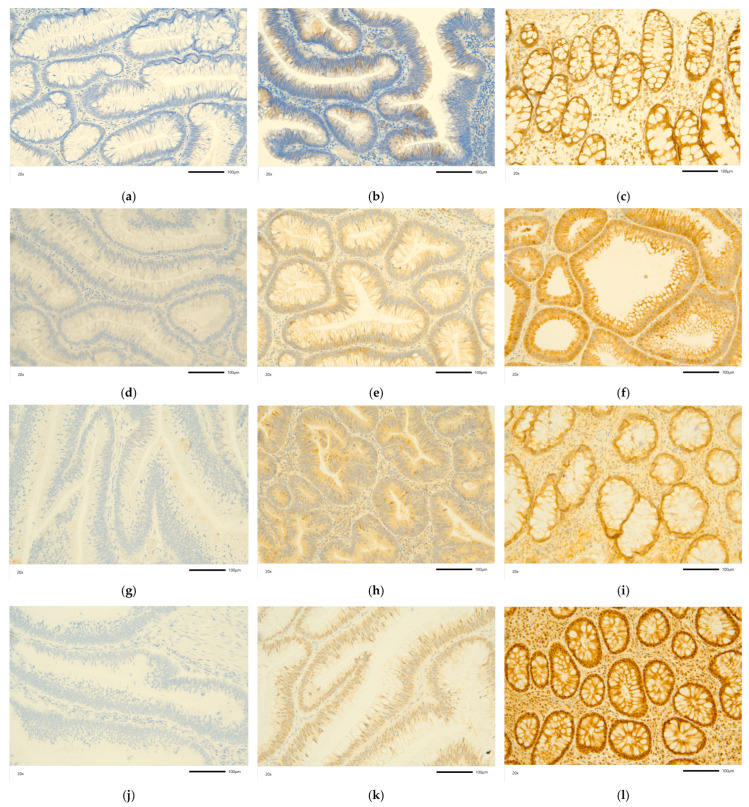
Immunohistochemical analysis of MIP proteins in colorectal neoplasms: (**a**) CCL3 (–); (**b**) CCL3 (+); (**c**) CCL3 (++); (**d**) CCL4 (–); (**e**) CCL4 (+); (**f**) CCL4 (++); (**g**) CXCL2 (–); (**h**) CXCL2 (+); (**i**) CXCL2 (++); (**j**) CCL19 (-); (**k**) CCL19 (+); (**l**) CCL19 (++). Tissue slides were incubated with rabbit anti-human antibodies with a DAB chromogen, stained brown in the case of a positive reaction and counterstained with hematoxylin. Photos were taken at 20× and the scale is indicated by a bar below photos representing 100 µm.

Protein expression was semi-quantified using immunohistochemistry (IHC) in a subset of 62 patient-matched pairs of polyp and normal mucosa slides. Examples of negative or weak (−; score 0), moderately positive (+; score 1) and strongly positive (++; score 2) reactions for each analyzed chemokine, that is, CCL3, CCL4, CXCL2, and CCL19, are presented in Figure 6. 

#### 2.3.1. MIP Proteins in Polyps and Patient-Matched Normal Mucosa

In adenomas, only CCL4 was expressed at comparable level between the lesion and paired normal mucosa, while the expression of CCL3 (*p* = 0.006), CXCL2 (*p* = 0.023) and CCL19 (*p* = 0.016) was significantly higher in lesions. In hyperplastic polyps, the visibly different expression of CCL4, lower in polyp than normal tissue, and of CCL3, higher in polyp than normal tissue, did not reach statistical significance as only four samples were available for analysis (Table S4).

**Figure 7:**
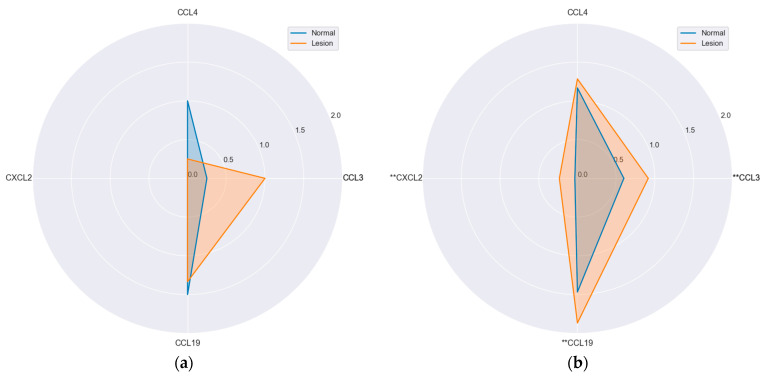
Protein expression of MIP chemokines in polyps and patient-matched normal mucosa: (**a**) hyperplastic polyps; (**b**) adenomas. Radar plots of observed mean rank values of immunoreactivity with “−, +, ++” categories rescaled to “0, 1, 2”. **, statistically significant.

Hyperplastic polyps differed from adenomas with significantly higher CCL19 (*p* = 0.017) and CCL4 (*p* = 0.007) expression in lesions (Figure 7). 

#### 2.3.2. MIP Proteins and Histological Type of Colorectal Neoplasms

**Figure 8:**
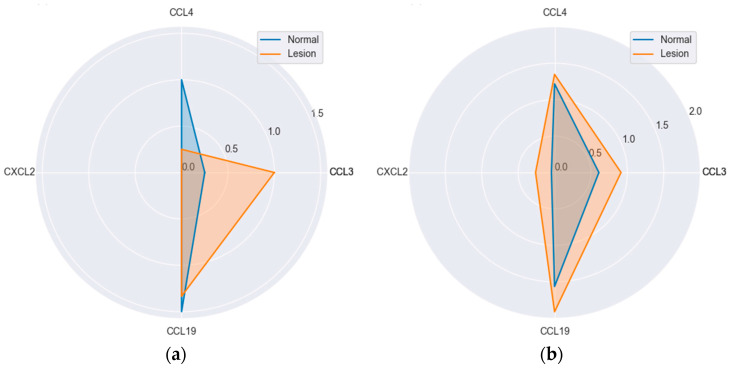

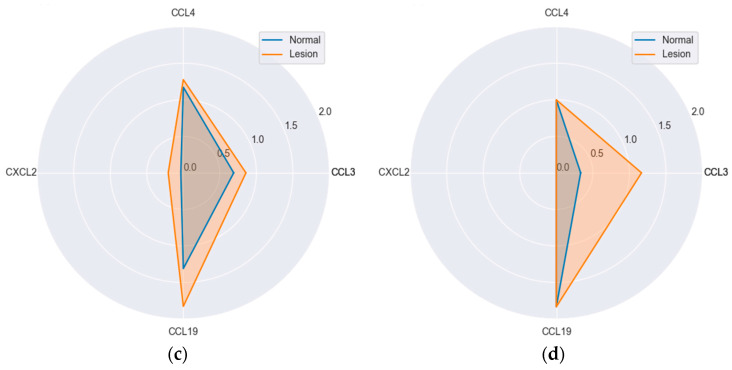
MIP protein expression in reference to polyp’s histological type: (**a**) hyperplastic polyps (H); (**b**) tubular adenomas (T); (**c**) tubulo-villous adenomas (T-V); (**d**) villous adenomas (V). Radar plots of observed mean rank values of immunoreactivity with “−, +, ++” categories rescaled to “0, 1, 2”.

Neither of chemokines differed significantly with respect to histological type either in lesions or normal mucosa. However, CCL4 (*p* = 0.052) and CCL19 (*p* = 0.088) expression in lesions tended to be lower in hyperplastic polyps (Figure 8). 

#### 2.3.3. MIP Proteins and Dysplasia Grade of Colorectal Neoplasms

CCL3 expression tended to depend on dysplasia grade. In normal tissue, it gradually increased from no dysplasia (H) through low- (LGD) to high-grade (HGD) dysplasia (*p* = 0.075). It was also slightly higher in HGD lesions.

CCL4 expression in lesions was comparable between LGD and HGD and significantly higher than in H (*p* = 0.031). In normal mucosa, it was comparable between H and LGD but was markedly upregulated in HGD (*p*_trend_ = 0.042).

CXCL2 expression in normal mucosa was negligible. In lesions it consequently, although slightly and non-significantly, increased from H through LGD to HGD.

**Figure 9:**
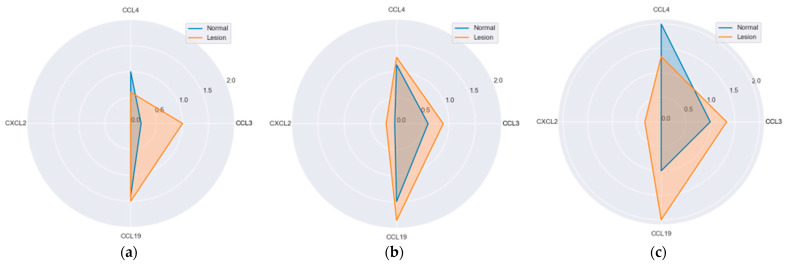
MIP protein expression in reference to dysplasia grade: (**a**) hyperplastic polyps (H); (**b**) adenomas with low grade dysplasia (LGD); (**c**) adenomas with high grade dysplasia (HGD). Radar plots of observed mean rank values of immunoreactivity with “−, +, ++” categories rescaled to “0, 1, 2”.

Likewise, CCL19 expression in lesions displayed an upward trend (*p*_trend_ = 0.027) while its expression in normal mucosa was significantly lower in HGD than LGD (*p* = 0.022) (Figure 9).

#### 2.3.4. MIP Proteins and Size of Colorectal Neoplasms

**Figure 10:**
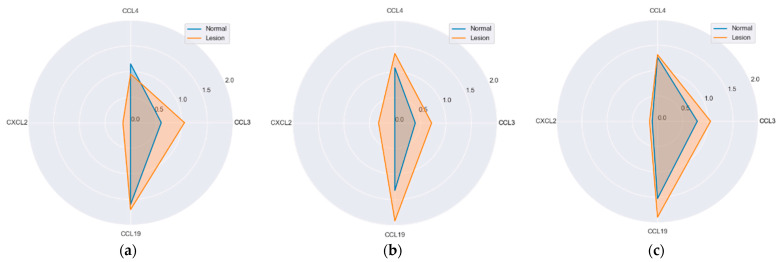
MIP protein expression in reference to polyp size: (**a**) polyps of <10 mm (S); (**b**) polyps of 10–19 mm (M); (**c**) polyps of ≥20 mm (L). Radar plots of observed mean rank values of immunoreactivity with “−, +, ++” categories rescaled to “0, 1, 2”.

CCL3 expression was lower in middle-sized polyps (10–19 mm; M) as compared to small (<10 mm; S) and large (≥20 mm; L) polyps. This observation was statistically significant in lesions (*p* = 0.045) but non-significant in normal mucosa (*p* = 0.083) (Figure 10).

CCL4 expression in lesions was significantly lower in S than M and L polyps combined (*p* = 0.030). In normal mucosa, it was insignificantly lower in M polyps (Figure 10).

CXCL2 expression in normal mucosa was comparable between S and M polyps and, when co-analyzed, significantly lower than in L polyps (*p* = 0.026). In lesions, it was insignificantly higher in M polyps (Figure 10). 

CCL19 expression in lesions was comparable between M and L polyps and, when combined, significantly higher than in S (*p* = 0.033). In normal mucosa, it was insignificantly lower in M polyps (Figure 10).

#### 2.3.5. MIP Proteins and Polyp Sublocation in the Colorectum

**Figure 11:**
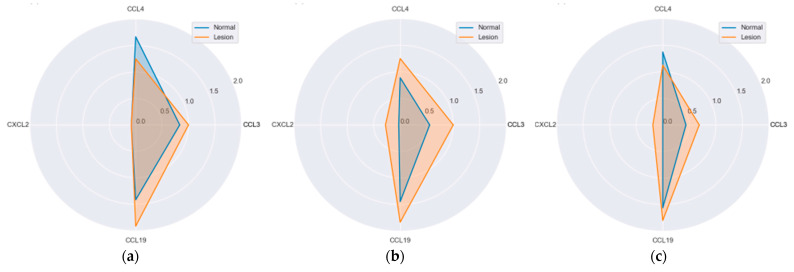
MIP protein expression in reference to polyp sublocation in the colorectum: (**a**) right colon; (**b**) left colon; (**c**) rectum. Radar plots of observed mean rank values of immunoreactivity with “−, +, ++” categories rescaled to “0, 1, 2”.

The expression of CCL4 in normal tissue depended on polyp sublocation (*p* = 0.015). It was the highest for right-sided colon (*p* = 0.032) an the lowest for left-sided colon (*p* = 0.004). In lesions, it was non-significantly higher in left-sided polyps yielding positive polyp-normal expression ratio (Figure 11).

Subtle differences in CCL3, CXCL2 and CCL19 expression illustrated in Figure 11 were non-significant both in lesions and normal mucosa and regardless of the testing approach, that is, whether they were analyzed as three separate sublocations (3 × 3 table) or as each sublocation against the other (2 × 3 tables).

## 3. Discussion

Studies on MIP chemokines in CRC are scarce, and those referring to precancerous lesions are even rarer. To the best of our knowledge, this is the first report in which MIP’s expression patterns were determined and compared between transcripts and proteins as well as between normal and transformed tissue with reference to standard malignancy risk factors in order to model the likelihood of cancerization and assess MIPs’ suitability as targets for chemoprevention. Importantly, this is a prospective study and the analyses were performed on a representative number of tissues. Corroborating previous findings [16,26,27,28] and results of dataset analysis [29], we observed a significant downregulation of *CCL3* and *CCL19* expression in polyps and an insignificant downregulation of *CCL4*. Unlike Hong et al. [26], but in line with the results of McLean et al. [16] and Doll et al. [28], *CXCL2* expression was upregulated in polyps and the reported magnitude of upregulation was consistent. In their unique study, Hong et al. [26] compared five sets of triplets—normal (N), adenoma (A), and adenocarcinoma (T) samples—obtained from the same patient. The authors presented the following chemokine expression patterns: N > A < T for *CCL3*, N = A < T for *CCL4* and *CXCL2*, and N > A = T for *CCL19*. Regarding CCL3, it was indeed overexpressed in CRC, both locally—mRNA [25,26] and protein [27,30]—and at a systemic level [31,32]. It reflects the disease advancement [27,31] and, in particular, lymph node involvement [25]. Functionally, CCL3 promotes cancer growth by stimulating proliferation, migration, and invasion, acting via the TRAF6/NF-κB pathway [27]. Moreover, it recruits cancer-associated fibroblasts (CAFs) and is highly expressed in tumor-associated macrophages (TAMs) [33]. Furthermore, CCL3 has been reported to induce angiogenesis [34] and facilitate osteolytic bone metastases of several cancers [35]. Accordingly, targeting *CCL3* gene expression reduces colonic inflammation [36] and tumorigenesis in animal CRC models [37]. Like most cytokines, CCL3 is dual-natured and switches between contradictory activities depending on its cellular source and microenvironmental context [38]. Accordingly, CCL3 activities that hamper cancer growth have been also demonstrated. They are attributed to the chemokine impact on various populations of anti-tumor immune cells, including upregulating the number of CD45^+^ leukocytes in the tumor [39] and recruiting cytotoxic T and NK cells into the tumor microenvironment (TME) [40,41]. CCL4 is also upregulated in CRC [25,30] and its systematic concentrations are elevated [31,32]. The chemokine acts similarly to CCL3 with respect to being a promoter of cancer cell proliferation and survival [33] and a facilitator of metastasis [37,42]. In turn, the anti-tumor effects of CCL4 are associated with recruitment of CD103^+^ DCs [33], responsible for priming and activating CD8^+^ T cells recruited into TME [43].

Contrary to *CCL3* and *CCL4*, *CXCL2* expression was upregulated in pair-wised analysis, both at the transcript and protein level, although the protein expression was generally very low. Its average immunoreactivity score ranged from 0 in normal tissue to < 0.5 in lesions. There was also a clear elevation in *CXCL2* expression in normal and polyp tissue as well as in P/N value for polyps with adenocarcinomas, which, however, lacked statistical significance due to low sample number. This observation is in line with the finding that methylation of *CXCL2* is significantly reduced in colorectal tumors [44], which would alleviate a methylation-depended suppression of *CXCL2* transcription [45]. Indeed, *CXCL2* upregulation in CRC has been reported [25,28,29,46], although not unanimously [47], and found to reflect patients’ clinical outcome and CRC stage [48]. CXCL2 activates the NFκB pathway and NLRP3 inflammasome [14]. Elevated expression of this chemokine translates into tissue infiltration by pro-inflammatory and anticancer M1-polarized macrophages and the diminished presence of anti-inflammatory, protumor, and immunosuppressive M2-polarized macrophages [29]. Weaker positive correlations have been found between *CXCL2* and neutrophils and activated myeloid DCs, and CD4^+^ memory and Th2 T cells [29]. Contrary to M1 macrophages, recruiting other immune cells may support tumor growth. Accordingly, Th2 cells are implicated in tumor promotion, similarly to monocytic and granulocytic MDSC attracted by MIP-2α/CXCL2 [46,49]. Likewise, neutrophils recruited by the chemokine can be steered to differentiate into an immunosuppressive N2 phenotype [33,44,46]. Moreover, CXCL2 promotes cancer cell proliferation [14,50], migration and interaction with extracellular matrix (ECM) components [50], facilitating liver [14] and peritoneal [50] metastasis. Furthermore, the chemokine enables angiogenesis by inducing endothelial cells migration towards VEGF-A and by the concomitant upregulation of the growth factor at the tumor edge [14,33]. As a chemoattractant for myeloid-derived suppressor cells (MDSCs), by which it is also actively secreted, CXCL2 inhibits the expansion and activity of anti-tumor immune cells, such as T and NK cells [51,52] and partakes in cancer metastasis by preparing metastatic niche, escorting circulating tumor cells, and enabling their extravasation [52].

Regarding CCL19, overall chemokine downregulation has been reported in CRC at both the protein and mRNA level [25,26,53,54]. *CCL19* has been identified by dataset analyses as diagnostic marker, the downregulation of which has 90% accuracy in CRC detection [55]. Still, some authors have reported an upregulation of CCL19 at advanced CRC stages [25,46]. Others, in turn, have linked the chemokine overexpression with better prognosis, given its ability to recruit antitumor immune cells into TME [39,56]. Their presence translates into suppressed tumor growth [39,57], linked with an upregulation of IFNγ and IL-12 secretion [57] and inhibition of angiogenesis [54].

Yamane et al. [8] noted that discerning molecular pathways leading from benign to malignant lesion was a prerequisite to improve risk stratification necessary to optimize screening or surveillance strategies; therefore, MIP expression was analyzed with respect to polyp’s characteristics. Most sporadic CRCs progress via an adenoma-carcinoma sequence but up to 30% develop from serrated polyps [58], of which hyperplastic polyps constitute up to 90% [8]. Dominant genetic and epigenetic events in the serrated pathway are *BRAS* or *KRAS* mutations and a CpG island methylator phenotype (CIMP), respectively. Clinically, the serrated pathway is a main contributor to post-colonoscopy (interval) CRC with a propensity to metachronous and synchronous cancers [59], thereby necessitating the search for molecular markers allowing for malignancy risk stratification [8,59]. Of the chemokines evaluated herein, a pathway of cancerization significantly affected *CCL3* expression. Consistent with an observation on gene elevation in inflammatory hyperplasia [27], *CCL3* was upregulated in hyperplastic polyps and downregulated in classic adenomas. Likewise, CCL19 and CCL4 proteins were significantly more abundant in hyperplastic polyps than adenomas. Polyp histology affected *CCL3, CCL4* and *CCL19* expression, which was significantly lower in polyps with at least 50% of villous growth pattern (T-V and V), an acknowledged risk factor for malignant transformation [60]. In a follow-up of patients diagnosed with polyps and reference individuals from general population, a 10-year cumulative CRC incidence was calculated at 1.6% for hyperplastic polyps and at 2.6% for tubular adenomas as compared to 2.1% in general population. It was, however, substantially higher in tubulo-villous and villous adenomas, calculated at 5.1% and 8.6%, respectively [61]. Taken together, our results might imply a protective role for *CCL3*, *CCL4* and *CCL19* in precancerous lesions. This notion is substantiated by *CCL19* downregulation in adenomas with high-grade dysplasia and by an inverse association of *CCL3* with polyp size and of *CCL4* with number of polyps. High-grade dysplasia and polyp size ≥ 20 mm are independent risk factors for CRC incidence after polyp removal [62]. Evidence that the size matters has been confirmed by an observation that no cancers are found in diminutive (<5 mm) and small (6–9 mm) adenomas [63,64]. Consistently, adenocarcinomas are increasingly detected in larger polyps, constituting 1.2% of cases in 10–19 mm category and 6.9% in ≥20 mm category [65]. Regarding the fourth risk factor, Wieszczy et al. [62] did not find number of polyps be an independent CRC predictor following polyp removal. Others, however, have shown the incidence of metachronous high-risk (MHR) neoplasms to rise gradually from 9.9% for 1–2 polyps removed and 57.1% for over 10 polyps removed during colonoscopy. Moreover, having removed at least 5 polyps has been an independent predictor of MHR colorectal neoplasms with odds ratio of 2.6 when adjusted to other factors [66].

As commented by Patel et al. [9], understanding the mechanism of transformation requires the analysis of tissue that has not yet been changed, and not that in which the transformation has already taken place. The notion has been substantiated by numerous observations on alterations in molecular pathways in macroscopically and histologically normal tissue, adjacent not only to tumor resection margins [25,67] but also to polyps [68,69,70]. Moreover, these alterations are not random, as they reflect cancer or adenoma advancement. Herein, we addressed this issue and found that *CCL3*, *CCL4* and *CCL19* was depended also on the fourth risk factor, that is, number of polyps removed during colonoscopy. Genes’ expression dropped significantly in normal mucosa in the case of multiple polyps, what might substantiate the notion on protective role played by *CCL3*, *CCL4*, and *CCL19* in neoplastic transformation and imply that the goal of their downregulation in non-transformed mucosa is to prepare a permissive environment. Moreover, *CCL19* expression in non-transformed mucosa mirrored that in lesions in terms of its dependence on histological type and dysplasia grade as it was significantly lower in adenomas with dominant villous growth pattern or with carcinomas than in tubular adenomas and in higher- than lower-grade dysplasia. Regarding *CXCL2*, which did not display any significant relation to polyp characteristics if determined in lesions, we found its expression in non-transformed mucosa to correlate with three out of four polyp-associated risk factors, that is, histological type and size and dysplasia grade. Unlike in the case of other MIPs, *CXCL2* expression in normal mucosa increased with increasing proportion of the villous component in the polyp, was significantly higher in large polyps, also at protein level, and showed an upward trend along the sequence none—low-grade—high-grade dysplasia. CXCL2 is viewed as a cancer-supporting chemokine providing tumor cells with proliferative, survival, and angiogenic cues [71]. However, its role in the pre-malignant state, and especially in the untransformed mucosa surrounding the polyp, is unknown.

Proper malignancy risk assessment is of great importance as it serves to recommend endoscopic surveillance or screening and to establish surveillance schedule [72]. Too rigorous criteria lead to unnecessary endoscopies, burdening patients and health-care system, while criteria that are not stringent enough can delay cancer diagnosis and negatively affect patient’s prognosis. Therefore, further measures to nuance the risk of malignancy are needed and intensively sought [8]. Using probit regression, we modeled the cumulative probabilities of malignant transformation for a given risk factor, evaluating whether and how the baseline probability is affected by changes in MIPs’ expression. Importantly, results of these analyses confirm that cumulative probability of malignant transformation is shaped by gene expression not only in polyp but also in adjacent tissue and that risk of malignancy is further affected by their interplay.

Baseline probability associated with histological type, that is, a dominance of villous growth pattern, could be modified by changes in *CCL4* and *CXCL2*. CCL4 has been claimed a negative [33] as well as positive [73] prognosticator. We found that an increase in *CCL4* expression in a polyp reduces the baseline likelihood of malignancy. Thus, the decrease in *CCL4*, which was observed herein, increases the likelihood of malignancy, confirming a protective role for *CCL4* and supporting a claim on the chemokine being a positive prognosticator [73]. However, we also found that concomitant increase in normal adjacent tissue would reverse the relationship. Therefore, an increase in *CCL4* in normal mucosa and a decrease in polyp would reduce the probability, and an increase in *CCL4* in both tissues would increase the malignancy risk. Regarding *CXCL2*, its rising expression in normal mucosa adjacent to polyps with an increasing contribution of villous growth would reduce the likelihood of malignant transformation. This finding might imply a protective role of *CXCL2* in polyp’s environment at benign stages of neoplastic transformation. This finding is in line with observations of Yang et al. [29], who demonstrated that of the immune cells attracted by the chemokine, the strongest association was with proinflammatory and anti-tumor M1 macrophages, followed by immune cells of similar character, that is, neutrophils and myeloid DC. Consistently, a positive correlation between CXCL2 expression and overall survival has been reported [14,29].

Baseline probability associated with the presence of dysplasia and its grade was affected solely by *CCL3*, specifically, by ratio of its expression between polyp and non-transformed adjacent mucosa. Previously, CCL3 overexpression and/or oversecretion in CRC has been linked with both better [74] and worse [33,75] prognosis. In the precancerous stage, probit regression analysis indicated a greater cumulative risk of malignancy in the case of increasing P/N. Therefore, decreasing P/N observed herein would translate into reduction in baseline risk associated with dysplasia and its higher grade. Decreasing ratio might be a result of *CCL3* drop in polyps and/or an elevation in adjacent normal mucosa, which may be interpreted as a negative role played by chemokine in transformed tissue and positive in non-transformed tissue.

Baseline probability associated with polyp’s size was affected solely by *CXCL2*. Its increasing expression in adjacent tissue would decrease the likelihood of malignant transformation resulting from the size of neoplasm, further supporting the conclusion on positive role played by *CXCL2* in polyp’s microenvironment. The possible effect of MIPs’ expression on baseline probability associated with number of polyps removed during colonoscopy could not be modeled as there were only two categories for this ordinal state. Likewise, potential effect of chemokines on baseline probability of malignancy associated with polyp location was not investigated as the analysis requires categories to be ordered.

Data regarding potential impact of polyp’s sublocation on its malignant potential are equivocal. It has been claimed that polyps located in distal colon (sigmoid colon and rectum) are more likely to be malignant than polyps located in ascending to descending colon [76]. Still, larger polyps are more common in the right colon [77]. Thus, others have shown that while malignant polyps are the most common in the rectum, their frequency in the colon is higher in its right than left side. Moreover, polyp size affected the impact of location, as polyps of over 35 mm located in the rectum were less likely to contain carcinoma than polyps of the same size in the right or left colon (reviewed in [78]). Herein, of the evaluated chemokines, only *CCL3* and *CCL4* displayed differences in expression with respect to polyp location in the colorectum. The fold-change of *CCL3* was significantly higher in the case of right-sided than left-sided polyps, which was a consequence of changes in both lesions and non-transformed mucosa. *CCL4* was also higher in right-sided polyps than in other locations both when fold-change and lesion expression were analyzed. Previously we have established a dependence of circulating CCL3 and CCL4 on sublocation of primary tumor in the colorectum. Unlike local expression, their systemic concentrations were, respectively, the lowest and the highest in rectal CRC [79]. In turn, higher *CCL4* expression in the right-sided than left-sided location was confirmed on protein level, although in non-transformed adjacent mucosa. 

For a subset of patients, protein expression of chemokines was determined semi-quantitively and allowed us to find CCL4 abundance to depend on dysplasia and the polyp’s size in addition to location discussed above. In polyps, hyperplasia was characterized by higher amount of the chemokine than found in dysplasia, whether it was low- or high-grade dysplasia. In normal mucosa, in turn, dysplasia grade affected the CCL4 amount as the protein was more abundant in the tissue surrounding polyps with high-grade dysplasia. It was also more abundant in medium-sized and large polyps as compared to small ones. Like for *CCL4* gene expression, there was no significant difference in its protein amount when polyp-normal pairs were analyzed. Likewise, gene and protein expression of CXCL2 were consistent, showing both to be upregulated in lesions as compared to matched normal mucosa, in which, in turn, they were both upregulated in the case of large polyps. However, there were discrepancies between gene and protein expression of *CCL3* and *CCL19*, which were downregulated in polyps at mRNA but upregulated at protein level in pair-wise analysis. As results of relative expression are strongly affected by selection of normalizers, we used a pair of genes found to be stably expressed in the bowel under neoplastic conditions [80]. In addition, we checked the expressions against other normalizers, namely, *GAPDH* and *RN18*, and found them to yield consistent data regardless normalizers used and corroborating findings of others [16,26]. Some disparity could be explained by differences in techniques of protein and mRNA determination: IHC is cell-selective but semiquantitative while qPCR is fully quantitative but not cell-specific. Regarding CCL3 protein, we failed to confirm the associations with polyp’s characteristics observed for *CCL3* transcripts, except for its size. *CCL3* was gradually decreasing with increasing size, while CCL3 was significantly elevated in medium-sized polyps. Likewise, *CCL19* association with polyp’s histological type could not be confirmed for CCL19 protein. In turn, CCL19 depended on polyp size, being significantly more abundant in medium and large than small polyps. However, striking contraries were observed for *CCL19* transcripts and CCL19 proteins with respect to dysplasia. While *CCL19* was higher in low-grade than high-grade dysplasia in both normal and polyp tissue, CCL19 displayed an inverse pattern—it was higher in high-grade than low-grade dysplasia, also in both normal and polyp tissue. The transcript-protein disparity is not rare phenomenon as genome-wide studies show consistency in expression for ca. 40%, while 60% of variation in protein abundance is not explained by expression levels of their respective mRNAs [81]. It is attributed to regulatory mechanism at the post-transcriptional level. Accordingly, the neoplastic transformation of intestinal epithelium has been linked with an upregulation of the cell’s translational machinery, cumulatively enhancing the global capacity of translation with each driver mutation (*APC*, *SMAD4* and *TP53* loss and *KRAS* overexpression) acquired [82]. Moreover, chemokines’ stability at protein level might be increased, thereby prolonging their half-life. We have previously found a downregulated expression of chemokine atypical receptors (ACKRs) in our patients [68]. ACKRs act as chemokine decoys and facilitate chemokines’ degradation, specifically, CCL3 is a ligand for ACKR2 and CCL19 is a ligand for ACKR4 [83]. 

In summary, MIP chemokines display distinct expression patterns in neoplastic and normal mucosa depending on polyp histology and transformation advancement. Their evaluation may add to the assessment of cancerization probability associated with traditional risk factors and help in clinical decision making, e.g., in determining optimal surveillance colonoscopy schedules.

## 4. Materials and Methods

### 4.1. Specimen Acquisition and Further Pre-Analytical Processing

The study utilized tissue samples of the large intestine collected during medically indicated endoscopy from patients admitted into the Department of Minimally Invasive Surgery and Proctology or the Department of Gastroenterology and Hepatology of Wroclaw Medical University. Each lesion was sampled alongside with its respective counterpart of morphologically normal mucosa, located at a distance of ca. 10 cm. Afterwards, the specimen was labeled, according to the microscopic assessment after hematoxylin-eosin (HE) staining, by the Department of Clinical Pathology. Following pathological examination, samples from three patients, two with celiac disease and one with inflammatory polyp, were excluded. 

### 4.2. Patients

Inclusion criteria were as follows: full colonoscopy with polypectomy, age ≥ 18 yrs., informed consent, and no known cancer disease. Patients with hereditary colorectal cancer syndromes (hereditary non-polyposis colon cancer, familial adenomatous polyposis, Peutz–Jeghers syndrome, MUTYH-associated polyposis, etc.), family history of colorectal cancer, personal history of colorectal adenoma or adenocarcinoma, gastrointestinal diseases and conditions influencing the colorectal cancer risk (for instance inflammatory bowel disease such as Crohn’s disease or ulcerative colitis) were excluded. Characteristics of patients and polyps, as described previously [70], is presented in Table 10.

All procedures associated with sample collection were approved by the medical ethics committees of Wroclaw Medical University (decision no. KB-247/2018, dated 24 April 2018).

### 4.3. Quantification of Gene Expression with Use of Reverse-Transcribed Quantitative Polymerase Chain Reaction (RTqPCR)

Samples were thawed and homogenized in Fastprep 24 Homogenizer (MP Biomedical, OH, USA) with use of ceramic spheres and lysis buffer (PureLink™ RNA Mini Kit from Invitrogen, Thermo Fisher Scientific, Waltham, MA, USA) and β-mercaptoethanol (Sigma Aldrich, St. Luis, MO, USA) at 1:10 (*v*/*v*). The mass of tissue samples used for homogenization was up to 40 mg. Phenol-chloroform extraction was applied to isolate RNA. Subsequently, the samples were purified with use of PureLink™ RNA Mini Kit (Invitrogen) and digested with PureLink™ DNase Set (Invitrogen) to avoid contamination with DNA. A NanoDrop 2000 spectrophotometer (Thermo-Fisher Scientific) and LabChip microfluidic technology, using the Experion platform and Experion RNA StdSens analysis kits (BioRad, Herkules, CA, USA), were utilized in the assessment of quantity, quality, and integrity of isolated RNA.

Then, 1000 ng of the RNA (per sample) was transcribed to cDNA with use of iScript™ cDNA Synthesis Kit (BioRad) and C1000 thermocycler (BioRad).

Expression of target genes: *CCL3*, *CCL4*, *CXCL2*, and *CCL19* and normalizers *PPIA* and *RPLP0* was quantified in CFX96 Real-Time PCR system (BioRad), based on polymerase chain reaction (qPCR). In qPCR, 30 s of activation at 95 °C was followed by 40 cycles comprising of 5 s of denaturation at 95 °C, annealing and extension for 5 s at 61 °C. Melting curve analysis (60–95 °C, fluorescent readings every 0.5 °C) and agarose electrophoresis with SYBR Green detection were utilized to determine product specificity. The reaction mixture contained 1 µL of 10 nM forward (F) and reverse (R) target-specific and intron-spanning primers (provided by Genomed, Warsaw, Poland), 10 µL of 2×SsoFast EvaGreen^®^ Supermix (BioRad), 2 µL of 5-fold diluted (with water) cDNA template, and 6 µL of water. The primer sequences were as follows: 5′-ACTTTGAGACGAGCAGCCAGTG-3′ (F) 5′-TTTCTGGACCCACTCCTCACTG-3′ (R) for *CCL3* (amplicon size: 101 bp), 5′-GCTTCCTCGCAACTTTGTGGTAG-3′ (F) and 5′-GGTCATACACGTACTCCTGGAC-3′ (R) for *CCL4* (140 bp), 5′-GGCAGAAAGCTTGTCTCAACCC-3′ (F) and 5′-CTCCTTCAGGAACAGCCACCAA-3′ (R) for *CXCL2* (127 bp), 5′-AGCAGGAACCAAGCTTAGGCTG-3′ (F) and 5′-GGTGTCTTGTCCAGATGCTGCA-3′ (R) for *CCL19* (133 bp), 5′-GGCAAATGCTGGACCCAACACA-3′ (F) and 5′-TGCTGGTCTTGCCATTCCTGGA-3′ (R) for *PPIA* (161 bp), and 5′-TCACAACAAGCATACCAAGAAGC-3′ (F) and 5′-GTATCCGATGTCCACAATGTCAAG-3′ (R) for *RPLP0* (263 bp).

Validated primers′ sequences were proposed by Ori-Gene (Rockville, MD, USA; www.origene.com (accessed on 1 March 2021). Further information on pre-processing of the signal from RTqPCR and its standardization could be found in previous studies [25,68,69,70].

### 4.4. Evaluation of the Protein Content with Use of Immunohistochemical (IHC) Methods

Pre-processed, paraffin-embedded slices of tissue samples were bound to the microscope slides. Dewaxing of tissue fragments was based on conditioning them in high-pH buffer in 97 °C, with use of the PT Link system (Agilent Technologies Inc., Carpinteria, CA, USA). Subsequently, the samples were combined with Cusabio Technology LLC (Houston, TX, USA) rabbit polyclonal anti-human antibodies specific towards the respected chemokines (CCL3: cat. no. CSB-PA040299, CCL4: cat. no. CSBPA05949A0Rb, CXCL2: cat. no. CSB-PA08989A0Rb or CCL19: cat. no. CSB-PA962305). The aforementioned application of antibodies took place in the Autostainer Plus Link platform (Agilent Technologies Inc.). The detection of the effects of antibody conjugation was performed with the EnVision FLEX set (Agilent Technologies Inc.; cat. no. K800221-2) which utilizes 3,39-diaminobenzidine tetrahydrochloride (DAB) as a substrate for catalyzing the indicator reaction. For counterstaining, hematoxylin was used. Afterwards, the samples were analyzed under the Olympus System BX51 microscope with a U0TV.63XS digital camera (Olympus Corporation, Tokyo, Japan).

Granular cytoplasmic reaction occurred in all cases, as expected based on the antibodies’ characteristics. IHC scoring was based on the reaction in the cytoplasm of glandular epithelial cells. These cells were chosen for assessment since they form the mucous membrane of the large intestine in physiological and pathological conditions (i.e., in adenomas, regardless of their type). The scoring was assessed as shown below:

- Score 0 (denoted as −) was a negative result in which there was no reaction or the reaction occurred in the stromal area of the analyzed specimen;
- Score 1 (denoted as +) was a weak positive result, meaning that the cytoplasmic reaction in the glandular epithelial cells was associated with low intensity, or the reaction was observed only in a fraction of the specimen;
- Score 2 (denoted as ++) was a strong positive result which was associated with strong reaction intensity spotted in the entire sample.

### 4.5. Data Preprocessing and Statistical Analysis

Data preprocessing, statistical analysis and visualization were performed using (1) MedCalc^®^ Statistical Software version 22.016 (MedCalc Software Ltd., Ostend, Belgium; https://www.medcalc.org); (2) Python 3.9.13 with the following packages: numpy 1.23.0, pandas 1.4.3, matplotlib 3.6.0, seaborn 0.12.0, and scipy 1.9.3; (3) STATISTICA 13.0.

Data were tested for normality and homogeneity of variances using, respectively, Shapiro–Wilk and Levene tests. Depending on the normality of distribution, homogeneity of variances, and number of groups compared, between-group comparisons were performed with one of the following analyses: Kruskal–Wallis *H* test followed by Conover post hoc test or one-way ANOVA with Students-Neuman-Keuls post hoc analysis or tests based on *z*′ statistics with Bonferroni correction, Mann–Whitney *U* test, and *t*-test for independent samples. To analyze dependent samples, Wilcoxon test or *t*-test for paired samples was applied. To test for an ordered difference and its direction in medians, the Jonckheere–Terpstra test was applied. The *χ^2^* test and *χ^2^* test for trend, corrected with Benjamini–Hochberg method, were used in the case of ordinal data (contingency tables). The McNemar test was performed for ordinal data and dependent samples.

Ordinal probit regression (distribution: ordinal polynomial, link function: probit) was utilized for investigation whether the cumulative probability of encountering (among the tested population, denoted by probit *ϕ*) each set of states associated with lesion characteristics: type, dysplasia grade, and size, changes upon change in expression of *CCL3*, *CCL4*, *CXCL2*, and *CCL19*. The aforementioned probability was calculated with a reverse probit function. The models were set up to explore several features at once. It was assumed that each of the predicted states could be ordered by magnitude (e.g., A < B < C …, etc.). Each ordinal intercept of the model describes the baseline (cumulative) probability of encountering the set of states higher than referred to. The impact of each of the effects and their interactions on the cumulative probability was shown by the *ϕ* values: probability decreased if *ϕ* < 0, or increased if *ϕ* > 0. The models were derived in a classic, reverse algorithm. Non-significant factors were discarded, stepwise, in the following order: normal × polyp × (P/N), P/N, and normal × polyp. The base features (polyp and normal NRQs) were always kept in the model.

## 5. Conclusions

The expression of MIP chemokines is altered in colorectal mucosa of patients with benign neoplasms, reflecting polyp advancement in terms of malignancy risk factors, such as histological type, the presence and grade of dysplasia, lesion size, and number of polyps removed during polypectomy. Normal tissue is an equal participant in carcinogenesis compared to transformed tissue as changes in chemokines’ expression associated with polyp characteristics occur either in lesions, morphologically and histologically normal polyp-adjacent mucosa, or in both. MIP chemokines modulate the likelihood of malignancy conferred by traditional risk factors and can help in polyp sub-stratification within the same malignancy risk group. CCL3 and CCL4 proteins are inversely expressed than their corresponding mRNAs, which should be taken into consideration while devising chemotherapies based on these chemokines.

## Figures and Tables

**Table 1:** Fold-change in *CCL3*, *CCL4*, *CXCL2*, and *CCL19* expression in colorectal neoplasms.

Gene	Fold-Change in Expression (P/N)—Pair-Wise Analysis				P/N Comparison across Groups
	All Cases	H	A	AC	
*CCL3*	↓1.7 (1.3–2.2) ^2,3^	↑2.5 (1–6.2) ^1,4,^*	↓1.8 (1.4–2.4) ^2,4,^*	↓3.7 (0.1–137) ^4^	*p* = 0.008 ^5^
*CCL4*	↓1.4, (1.3–1.5) ^3^	↑1.2 (0.4–3.8) ^4^	↓1.4 (1.3–1.4) ^3^	↓3.6 (0.2–64) ^4^	*p* = 0.373 ^6^
*CXCL2*	↑2.5 (2.2–2.5) ^2,3^	↑2.3 (0.9–5.7) ^4^	↑2.5 (2.2–2.5) ^2,3^	↑2.1 (0–126) ^4^	*p* = 0.891 ^6^
*CCL19*	↓3.1, (2.1–4.7) ^2,4^	↓1.3 (0.3–5.6) ^4^	↓3.2 (2.2–4.8) ^2,3^	↓21.4 (0.1–5600) ^4^	*p* = 0.167 ^5^

Data presented as means or medians with 95% *CI* and analyzed by tests for paired samples (in the pair-wise analysis section) or by *p* value for multigroup comparisons (in the last column; comparison of mean/median P/N in H vs. A vs. AC groups). The *p* values for pair-wise testing is indicated by superscript numbers as follows: ^1^, *p* < 0.05; ^2^, *p* < 0.001. Statistical tests were applied based on data distribution and homogeneity of variances and indicated by the superscript number as follows: ^3^, Wilcoxon test; ^4^, *t*-test for paired samples; ^5^, one-way ANOVA; ^6^, Kruskal–Wallis *H* test; *, significantly different from each other. P/N, polyp-to normal ratio; H, hyperplasic polyp (serrated pathway); A, adenoma; AC, adenocarcinoma in polyp; *CI*, confidence interval; ↓, relative downregulation in polyp; ↑, relative upregulation in polyp.

**Table 2:** Effect of polyp histological type on fold-change in chemokine expression.

Gen	Fold-Change in Gene Expression (P/N)					*p* ^1^	*p* ^2^
	H	T	T-V	V	AC ^4^		
*CCL3*	↑2.5 (1.2–6.9)	↓1.4 ^3^ (1.0–2.0)	↓2.0 ^3^ (1.3–2.7)	↓2.8 ^3^ (0.6–25)	↓2.8 ^3^ (1.1–41)	0.015	0.381
*CCL4*	↑1.5 (0.4–3.8)	1.0 (0.8–2.7)	↓1.1 (0.9–1.7)	↓1.9 (0.5–9.5)	↓4.7 (0.9–19)	0.512	0.527
*CXCL2*	↑2.3 (1.4–8.5)	↑1.4 (1.1–3.2)	↑2.3 (1.8–3.3)	↑1.2 (0.2–3.3)	↑5.4 (0.5–18.5)	0.461	0.167
*CCL19*	↑1.4 (0.2–5.1)	↓3.5 (0.7–6.5)	↓3.9 (1.7–5.5)	↓22.2 (0.6–57.5)	↓27.8 (13–609)	0.240	0.360

Data were analyzed using Kruskal–Wallis *H* test and are presented as medians with 95% *CI*. *CI*, confidence interval; P/N, polyp-to-normal ratio; H, hyperplastic polyp; T, tubular adenoma; T-V, tubulo-villous adenoma; V, villous adenoma; AC, carcinoma in the polyp; ↓, relative downregulation in polyp; ↑, relative upregulation in polyp; ^1^, probability for all analyzed histological types; ^2^, probability for analysis restricted to adenomas; ^3^, significantly different from hyperplastic polyps; ^4^, presented as medians with interquartile range (sample too small for *CI* calculation).

**Table 3:** Effect of dysplasia grade on fold-change in chemokine expression.

Gene	Fold-Change in Gene Expression (P/N)			*p* ^1^	*p* ^2^
	H	LGD	HGD		
*CCL3*	↑2.5 (1.2–6.9)	↓1.8 (1.3–2.5) ^3^	↓1.4 (0.8–3.4) ^3^	0.006	0.930
*CCL4*	↑1.5 (0.4–3.8)	↓1.1 (0.9–1.6)	↓1.1 (0.6–2.4)	0.672	0.879
*CXCL2*	↑2.3 (1.3–8.5)	↑2.0 (1.6–3.2)	↑2.1 (1.6–3.1)	0.859	0.706
*CCL19*	↑1.4 (0.2–5.1)	↓4.6 (2.6–5.7)	↓1.7 (0.6–23.9)	0.357	0.581

Data were analyzed using Kruskal–Wallis *H* test (*p* ^1^) or Mann–Whitney *U* test (*p* ^2^) and are presented as medians with 95% *CI*. ^1^, probability for all analyzed categories; ^2^, probability for analysis restricted to dysplasia; ^3^, significantly different from hyperplasia; P/N, polyp-to-normal ratio; H, hyperplasia; LGD, low-grade dysplasia; HGD, high-grade dysplasia; *CI*, confidence interval; ↓, relative downregulation in polyp; ↑, relative upregulation in polyp.

**Table 4:** Effect of polyp size on fold-change in chemokine expression.

Gene	Fold-Change in Gene Expression (P/N)			*p*
	≤10 mm	10–19 mm	≥20 mm	
*CCL3*	↓1.3 (0.8–2.5)	↓1.7 (1–2.5)	↓2.0 (1.1–3.3)	0.468
*CCL4*	1.0 (0.8–1.7)	↓1.2 (0.8–1.8)	↓1.4 (0.8–2.5)	0.429
*CXCL2*	↑1.8 (1.4–2.3)	↑2.7 (1.8–3.9)	↑1.8 (1.1–3.1)	0.091
*CCL19*	↓2.9 (0.7–5.5)	↓4.6 (0.9–7.4)	↓3.9 (1.7–12.4)	0.703

Data were analyzed using Kruskal–Wallis *H* test and presented as medians with 95% *CI*. *CI*, confidence interval; P/N, polyp-to-normal ratio; ↓, relative downregulation in polyp; ↑, relative upregulation in polyp.

**Table 5:** Effect of number of polyps and their character on fold-change in chemokine expression.

Gene	Fold-Change in Gene Expression (P/N)			*p*
	One	Multiple	Carpet-like	
*CCL3*	↓1.8 (1.3–2.3)	↓1.2 (0.7–3.8)	↑1.9 (0.1–4.9)	0.504
*CCL4*	↓1.2 (1–1.7)	↓1.1 (0.4–2.8)	↑2.8 (0.1–8.1)	0.462
*CXCL2*	↑2.0 (1.6–2.8)	↑2.1 (1.4–3.2)	↑3.2 (0.1–19.9)	0.987
*CCL19*	↓4.4 (2.3–5.6)	↓1.8 (0.3–8.2)	↓1.5 (0.1–893)	0.303

Data were analyzed using Kruskal–Wallis *H* test and presented as medians with 95% *CI*. *CI*, confidence interval; P/N, polyp-to-normal ratio; ↓, relative downregulation in polyp; ↑, relative upregulation in polyp.

**Table 6:** Effect of polyps sublocation in the colorectum on fold-change in chemokine expression.

Gene	Fold-Change in Gene Expression (P/N)				*p* ^1^	*p* ^2^
	Right Colon	Left Colon	Rectum	Distal Colon		
*CCL3*	↑1.2 (0.7–1.8)	↓1.9 (1.3–3.1) ^3^	↓2.0 (0.7–2.7)	↓1.9 (1.4–2.5)	0.041	0.016
*CCL4*	↑1.5 (0.8–2.7)	↓1.4 (1–2.5)	↓1.2 (0.7–1.9)	↓1.3 (1–1.8)	0.063	0.025
*CXCL2*	↑2.5 (1.6–4.2)	↑2.0 (1.6–2.5)	↑2.2 (1.3–3.4)	↑2.0 (1.6–2.6)	0.536	0.271
*CCL19*	↓1.6 (0.5–4.8)	↓4.2 (1.9–7)	↓5.2 (1.5–19.9)	↓4.6 (2.7–7)	0.242	0.127

Data are presented as medians with 95% *CI*. ^1^, probability for Kruskal–Wallis *H* test; ^2^, probability for Mann–Whitney *U* test analysis of right colon vs. distal colon (left colon and rectum); ^3^, significantly different from right colon; *CI*, confidence interval; P/N, polyp-to-normal ratio; ↓, relative downregulation in polyp; ↑, relative upregulation in polyp.

**Table 7:** Cumulative probabilities for histological type as predicted ordinal state.

Feature	*CCL3*		*CCL4*		*CXCL2*		*CCL19*	
	*ϕ_i_*	*p*	*ϕ_i_*	*p*	*ϕ_i_*	*p*	*ϕ_i_*	*p*
Intc. 1 (pred. state: AC)	−1.81	<0.001	−1.61	<0.001	−1.46	<0.001	−1.62	<0.001
Intc. 2 (pred. state: V ∨ AC)	−0.77	<0.001	−0.62	<0.001	−0.49	<0.001	−0.62	<0.001
Intc. 3 (pred. state: TV ∨ V ∨ AC)	1.13	<0.001	1.25	<0.001	1.38	<0.001	1.25	<0.001
Intc. 4 (pred. state: T ∨ TV ∨ V ∨ AC)	1.86	<0.001	1.98	<0.001	2.13	<0.001	1.97	<0.001
Expression in normal mucosa	−0.004	0.146	−0.01	0.091	−0.061	0.023 *	−0.01	0.245
Expression in polyp (lesion)	0.03	0.550	−0.024	0.020 *	−0.01	0.583	−0.01	0.507
P/N	0.07	0.145	-	0.105	-	0.807	-	0.616
Normal × polyp × P/N	−0.0003	0.012 *	-	0.622	-	0.387	-	0.134
Normal × polyp	0.01	0.470	0.025	0.011 *	-	0.963	0.005	0.011 *

Missing *ϕ*_i_ values indicate that the feature was excluded from the model in the stepwise process of its derivation. *ϕ_i_*, probit function (cumulative probability); intc., intercept; pred., predicted; AC, adenocarcinoma in the polyp; V, villous adenoma; TV, tubolo-villous adenoma; T, tubular adenoma; H, hyperplastic polyp; ∨, “or”; P/N, polyp-to-normal ratio. Statistically significant effects are indicated by * (asterisks).

**Table 8:** Cumulative probabilities for dysplasia grade as predicted ordinal state.

Feature	*CCL3*		*CCL4*		*CXCL2*		*CCL19*	
	*ϕ_i_*	*p*	*ϕ_i_*	*p*	*ϕ_i_*	*p*	*ϕ_i_*	*p*
Intc. 1 (pred. state: AC)	−1.88	<0.001	−1.55	<0.001	−1.48	<0.001	−1.64	<0.001
Intc. 2 (pred. state: HG ∨ AC)	0.67	<0.001	0.85	<0.001	0.94	<0.001	0.79	<0.001
Intc. 3 (pred. state: LG ∨ HG ∨ AC)	1.79	<0.001	1.95	<0.001	2.05	<0.001	1.90	<0.001
Expression in normal mucosa	0.00	0.847	0.00	0.908	−0.04	0.101	0.00	0.519
Expression in polyp (lesion)	0.01	0.930	0.00	0.449	−0.00	0.785	0.02	0.108
P/N	0.123	0.033 *	-	0.839	-	0.736	-	0.870
Normal × polyp × P/N	−0.0004	0.004 *	-	0.133	-	0.550	-	0.419
Normal × polyp	0.01	0.539	-	0.118	-	0.223	-	0.068

Missing *ϕ*_i_ values indicate that the feature was excluded from the model in the stepwise process of its derivation. *ϕ_i_*, probit function (cumulative probability); intc., intercept; pred., predicted; AC, adenocarcinoma in the polyp; HG, high-grade dysplasia; LG, low-grade dysplasia; ∨, “or”; P/N, polyp-to-normal ratio. Statistically significant effects are indicated by * (asterisks).

**Table 9:** Cumulative probabilities for polyp size as predicted ordinal state.

Feature	*CCL3*		*CCL4*		*CXCL2*		*CCL19*	
	*ϕ_i_*	*p*	*ϕ_i_*	*p*	*ϕ_i_*	*p*	*ϕ_i_*	*p*
Intc. 1 (pred. state: L)	−0.73	<0.001	−0.72	<0.001	−0.57	<0.001	−0.8176	<0.001
Intc. 2 (pred. state: M ∨ L)	0.46	<0.001	0.47	<0.001	0.66	<0.001	0.3926	<0.001
Expression in normal mucosa	−0.01	0.334	−0.01	0.243	−0.194	0.005 *	−0.00	0.724
Expression in polyp	0.00	0.838	0.00	0.845	0.00	0.838	0.03	0.067
P/N	-	0.333	-	0.817	-	0.269	-	0.388
Normal × polyp × P/N	-	0.185	-	0.942	-	0.747	-	0.749
Normal × polyp	-	0.320	-	0.439	-	0.829	-	0.494

Missing *ϕ*_i_ values indicate that the feature was excluded from the model in the stepwise process of its derivation. *ϕ_i_*, probit function (cumulative probability); intc., intercept; pred., predicted; L, large: polyp of ≥ 20 mm; M, moderate: polyp of 10–19 mm; S, small: polyp of <10 mm; ∨, “or”; P/N, polyp-to-normal ratio. Statistically significant effects are indicated by * (asterisks).

**Table 10:** Characteristics of patients and polyps.

Parameter	Protein Analysis	Gene Expression Analysis
*n*	62	173
Sex distribution: F/M, *n*	27/35	78/95
Age [yrs.], mean (95% *CI*)	62.9 (60.2–65.7)	65.3 (63.6–67.0)
Weight, *n*:		
Lean/Overweight/Obese/x	29/27/6/0	77/74/20/2
Smoking status, *n*:		
no/yes/x	40/22/0	120/51/2
Alcohol, *n*:		
no/occasional/moderate/AUD/x	3/58/1/0/0	15/151/4/1/2
Type 2 diabetes, *n*:		
no/yes/x	49/13/0	135/36/2
Arterial hypertension, *n*:		
no/yes/x	19/43/0	64/107/2
Polyp histology, *n*:		
hyperplastic polyps	4	11
tubular adenoma	23	37
tubulo-villous adenoma	29	107
villous adenoma	6	13
adenocarcinoma in the polyp	0	5
Grade of dysplasia, *n*:		
low/high	55/3	128/29
Polyp size, *n*:		
<10 mm/10–19 mm/≥20 mm	19/24/19	39/75/58
Polyp location, *n*:		
right colon/left colon/rectum	12/35/15	90/38/45
Number of polyps, *n*:		
Single/multiple (≥2)	60/2	129/36
carpet-like lesions	0	7

*n*, number of observations; F/M, female-to-male ratio; yrs., years; *CI*, confidence interval; x, missing data; AUD, alcohol use disorder.

## Data Availability

Dataset available on request from the authors.

## Supplementary Materials

The supporting information can be downloaded at: https://www.mdpi.com/article/10.3390/ijms25031383/s1.
