# Supplementary material for: Macrophage Inflammatory Proteins (MIPs) Contribute to Malignant Potential of Colorectal Polyps and Modulate Likelihood of Cancerization Associated with Standard Risk Factors
Source: Int J Mol Sci. 2024 Jan 23;25(3):1383. doi: 10.3390/ijms25031383 (PMC10855842; doi:10.3390/ijms25031383)
Supplement: Supplementary file 1 [file ijms-25-01383-s001.zip › ijms-2788651-supplementary.pdf]

Supplementary Material

Macrophage Inflammatory Proteins (MIPs) Contribute to Malignant Potential of Colorectal Polyps and Modulate Likelihood of Cancerization Associated with Standard Risk Factors

Jarosław Wierzbicki, Iwona Bednarz-Misa, Łukasz Lewandowski, Artur Lipiński, Anna Kłopot, Katarzyna Neubauer and Małgorzata Krzystek-Korpacka

Table S1. Cumulative probabilities for histological type as predicted ordinal state

Gene: CCL3							
**Feature**	**ϕi**	**Φi SD**	**Wald statistics**	**Φi 95% CI**	**Φi 95% CI**	**Predicted probability**	**p-value**
Intercept 1 (predicted state: AC)	-1.8111	0.1873	93.489	-2.1782	-1.4440	0.035	<0.001
Intercept 2 (predicted states: V or AC)	-0.7650	0.1264	36.616	-1.0128	-0.5172	0.222	<0.001
Intercept 3 (predicted states: TV or V or AC)	1.1301	0.1458	60.078	0.8443	1.4158	0.871	<0.001
Intercept 4 (predicted states: T or TV or V or AC)	1.8638	0.2245	68.939	1.4238	2.3037	0.969	<0.001
CCL3 normal	-0.0044	0.0030	2.118	-0.0103	0.0015	-	0.146
CCL3 lesion	0.0299	0.0500	0.358	-0.0681	0.1279	-	0.550
CCL3 lesion/normal	0.0741	0.0509	2.119	-0.0257	0.1738	-	0.145
CCL3 normal×CCL3 lesion×CCL3 lesion/normal	-0.0003	0.0001	6.263	-0.0006	-0.0001	-	0.012
CCL3 normal×CCL3 lesion	0.0078	0.0109	0.521	-0.0134	0.0291	-	0.470
**Gene: CCL4**							
**Feature**	**ϕi**	**Φi SD**	**Wald statistics**	**Φi 95% CI**	**Φi 95% CI**	**Predicted probability**	**p-value**
Intercept 1 (predicted state: AC)	-1.605	0.1654	94.163	-1.9292	-1.2808	0.054	<0.001
Intercept 2 (predicted states: V or AC)	-0.615	0.1112	30.593	-0.8327	-0.3970	0.269	<0.001
Intercept 3 (predicted states: TV or V or AC)	1.251	0.1386	81.454	0.9793	1.5226	0.895	<0.001
Intercept 4 (predicted states: T or TV or V or AC)	1.982	0.2206	80.755	1.5498	2.4144	0.976	<0.001
CCL4 normal	-0.010	0.0061	2.864	-0.0222	0.0016	-	0.091
CCL4 lesion	-0.024	0.0103	5.412	-0.0440	-0.0038	-	0.020
CCL4 lesion/normal	-	-	-	-	-	-	0.105
CCL4 normal×CCL4 lesion×CCL4 lesion/normal	-	-	-	-	-	-	0.622

Feature	ϕi	Φi SD	Wald statistics	Φi 95% CI	Φi 95% CI	Predicted probability	p-value
CCL4 normal×CCL4 lesion	0.025	0.0098	6.428	0.0057	0.0441	-	0.011
**Gene: CXCL2**							
**Feature**	**ϕi**	**Φi SD**	**Wald statistics**	**Φi 95% CI**	**Φi 95% CI**	**Predicted probability**	**p-value**
Intercept 1 (predicted state: AC)	-1.4610	0.1614	81.960	-1.7773	-1.1447	0.072	<0.001
Intercept 2 (predicted states: V or AC)	-0.4913	0.1132	18.827	-0.7133	-0.2694	0.312	<0.001
Intercept 3 (predicted states: TV or V or AC)	1.3745	0.1469	87.591	1.0866	1.6623	0.915	<0.001
Intercept 4 (predicted states: T or TV or V or AC)	2.1279	0.2340	82.668	1.6692	2.5867	0.983	<0.001
CXCL2 normal	-0.0614	0.0271	5.133	-0.1146	-0.0083	-	0.023
CXCL2 lesion	-0.0048	0.0088	0.301	-0.0220	0.0124	-	0.583
CXCL2 lesion/normal	-	-	-	-	-	-	0.807
CXCL2 normal×CXCL2 lesion×CXCL2 lesion/normal	-	-	-	-	-	-	0.387
CXCL2 normal×CXCL2 lesion	-	-	-	-	-	-	0.963
**Gene: CCL19**							
**Feature**	**ϕi**	**Φi SD**	**Wald statistics**	**Φi 95% CI**	**Φi 95% CI**	**Predicted probability**	**p-value**
Intercept 1 (predicted state: AC)	-1.6216	0.1722	88.675	-1.9592	-1.2841	0.052	<0.001
Intercept 2 (predicted states: V or AC)	-0.6196	0.1185	27.354	-0.8518	-0.3874	0.268	<0.001
Intercept 3 (predicted states: TV or V or AC)	1.2466	0.1438	75.164	0.9648	1.5284	0.894	<0.001
Intercept 4 (predicted states: T or TV or V or AC)	1.9731	0.2211	79.603	1.5397	2.4065	0.976	<0.001
CCL19 normal	-0.0066	0.0057	1.354	-0.0177	0.0045	-	0.245
CCL19 lesion	-0.0083	0.0125	0.441	-0.0328	0.0162	-	0.507
CCL19 lesion/normal	-	-	-	-	-	-	0.616
CCL19 normal×CCL19 lesion×CCL19 lesion/normal	-	-	-	-	-	-	0.134
CCL19 normal×CCL19 lesion	0.0046	0.0018	6.466	0.0010	0.0081	-	0.011

SD, standard deviation; CI, confidence interval; AC, adenocarcinoma in the polyp; V, villous adenomas; TV, tubulo-villous adenomas; T, tubular adenomas

Table S2. Cumulative probabilities for dysplasia grade as predicted ordinal state

Gene: CCL3							
**Feature**	**ϕi**	**Φi SD**	**Wald statistics**	**Φi 95% CI**	**Φi 95% CI**	**Predicted probability**	**p-value**
Intercept 1 (predicted state: AC)	-1.8769	0.2006	87.521	-2.2701	-1.4837	0.030	<0.001
Intercept 2 (Predicted states: HGD or AC)	0.6741	0.1276	27.911	0.4240	0.9241	0.750	<0.001
Intercept 3 (Predicted states: LGD, HGD or AC)	1.7886	0.2186	66.923	1.3600	2.2171	0.963	<0.001
CCL3 normal	0.0007	0.0034	0.037	-0.0060	0.0073	-	0.847
CCL3 lesion	0.0046	0.0522	0.008	-0.0976	0.1068	-	0.930
CCL3 lesion/normal	0.123	0.0577	4.524	0.0096	0.2359	-	0.033
CCL3 normal×CCL3 lesion×CCL3 lesion/normal	-0.0004	0.0001	8.165	-0.0007	-0.0001	-	0.004
CCL3 normal×CCL3 lesion	0.0076	0.0123	0.378	-0.0166	0.0317	-	0.539

Gene: CCL4							
**Feature**	**ϕi**	**Φi SD**	**Wald statistics**	**Φi 95% CI**	**Φi 95% CI**	**Predicted probability**	**p-value**
Intercept 1 (predicted state: AC)	-1.5506	0.1599	94.095	-1.8639	-1.2373	0.060	<0.001
Intercept 2 (Predicted states: HGD or AC)	0.8471	0.1167	52.665	0.6183	1.0758	0.802	<0.001
Intercept 3 (Predicted states: LGD, HGD or AC)	1.9470	0.2131	83.453	1.5293	2.3647	0.974	<0.001
CCL4 normal	0.0007	0.0064	0.013	-0.0118	0.0132	-	0.908
CCL4 lesion	0.0034	0.0045	0.574	-0.0054	0.0122	-	0.449
CCL4 lesion/normal	-	-	-	-	-	-	0.839
CCL4 normal×CCL4 lesion×CCL4 lesion/normal	-	-	-	-	-	-	0.133
CCL4 normal×CCL4 lesion	-	-	-	-	-	-	0.118

Gene: CXCL2							
**Feature**	**ϕi**	**Φi SD**	**Wald statistics**	**Φi 95% CI**	**Φi 95% CI**	**Predicted probability**	**p-value**
Intercept 1 (predicted state: AC)	-1.4803	0.1613	84.222	-1.7964	-1.1641	0.069	<0.001
Intercept 2 (Predicted states: HGD or AC)	0.9356	0.1254	55.705	0.6899	1.1813	0.825	<0.001
Intercept 3 (Predicted states: LGD, HGD or AC)	2.0489	0.2243	83.411	1.6092	2.4886	0.980	<0.001

CXCL2 normal	-0.0427	0.0261	2.682	-0.0937	0.0084	-	0.101
CXCL2 lesion	-0.0025	0.0094	0.074	-0.0209	0.0158	-	0.785
CXCL2 lesion/normal	-	-	-	-	-	-	0.736
CXCL2 normal×CXCL2 lesion×CXCL2 lesion/normal	-	-	-	-	-	-	0.550
CXCL2 normal×CXCL2 lesion	-	-	-	-	-	-	0.223
**Gene: CCL19**							
**Feature**	**ϕi**	**Φi SD**	**Wald statistics**	**Φi 95% CI**	**Φi 95% CI**	**Predicted probability**	**p-value**
Intercept 1 (predicted state: AC)	-1.6352	0.1737	88.648	-1.9757	-1.2948	0.051	<0.001
Intercept 2 (Predicted states: HGD or AC)	0.7901	0.1247	40.119	0.5456	1.0346	0.785	<0.001
Intercept 3 (Predicted states: LGD, HGD or AC)	1.9038	0.2190	75.602	1.4747	2.3330	0.972	<0.001
CCL19 normal	0.0037	0.0058	0.417	-0.0076	0.0150	-	0.519
CCL19 lesion	0.0165	0.0103	2.582	-0.0036	0.0367	-	0.108
CCL19 lesion/normal	-	-	-	-	-	-	0.870
CCL19 normal×CCL19 lesion×CCL19 lesion/normal	-	-	-	-	-	-	0.419
CCL19 normal×CCL19 lesion	-	-	-	-	-	-	0.068

SD, standard deviation; CI, confidence interval; AC, adenocarcinoma in the polyp; HGD, high-grade dysplasia; LGD, low-grade dysplasia.

Table S3. Cumulative probabilities for polyp size as predicted ordinal state

**Gene: CCL3**							
**Feature**	**ϕi**	**Φi SD**	**Wald statistics**	**Φi 95% CI**	**Φi 95% CI**	**Predicted probability**	**p-value**
Intercept 1 (predicted state: ≥ 20 mm)	-0.7321	0.1122	42.562	-0.9520	-0.5121	0.232	<0.001
Intercept 2 (predicted states: 10 – 19 mm or ≥ 20 mm)	0.4624	0.1057	19.147	0.2553	0.6694	0.678	<0.001
CCL3 normal	-0.0057	0.0059	0.932	-0.0173	0.0059	-	0.334
CCL3 lesion	0.0005	0.0026	0.042	-0.0045	0.0056	-	0.838
CCL3 lesion/normal	-	-	-	-	-	-	0.333
CCL3 normal×CCL3 lesion×CCL3 lesion/normal	-	-	-	-	-	-	0.185
CCL3 normal×CCL3 lesion	-	-	-	-	-	-	0.320
**Gene: CCL4**							
**Feature**	**ϕi**	**Φi SD**	**Wald statistics**	**Φi 95% CI**	**Φi 95% CI**	**Predicted probability**	**p-value**
Intercept 1 (predicted state: ≥ 20 mm)	-0.7232	0.1138	40.414	-0.9461	-0.5002	0.235	<0.001
Intercept 2 (predicted states: 10 – 19 mm or ≥ 20 mm)	0.4723	0.1077	19.235	0.2612	0.6833	0.682	<0.001
CCL4 normal	-0.0102	0.0087	1.364	-0.0273	0.0069	-	0.243
CCL4 lesion	0.0008	0.0041	0.038	-0.0072	0.0088	-	0.845
CCL4 lesion/normal	-	-	-	-	-	-	0.817
CCL4 normal×CCL4 lesion×CCL4 lesion/normal	-	-	-	-	-	-	0.942
CCL4 normal×CCL4 lesion	-	-	-	-	-	-	0.439
**Gene: CXCL2**							
**Feature**	**ϕi**	**Φi SD**	**Wald statistics**	**Φi 95% CI**	**Φi 95% CI**	**Predicted probability**	**p-value**
Intercept 1 (predicted state: ≥ 20 mm)	-0.5729	0.1287	19.804	-0.8253	-0.3206	0.283	<0.001
Intercept 2 (predicted states: 10 – 19 mm or ≥ 20 mm)	0.6617	0.1292	26.227	0.4084	0.9149	0.746	<0.001
CXCL2 normal	-0.1940	0.0688	7.942	-0.3289	-0.0591	-	0.005
CXCL2 lesion	0.0018	0.0090	0.042	-0.0157	0.0194	-	0.838
CXCL2 lesion/normal	-	-	-	-	-	-	0.269

CXCL2 normal×CXCL2 lesion×CXCL2 lesion/normal	-	-	-	-	-	-	0.747
CXCL2 normal×CXCL2 lesion	-	-	-	-	-	-	0.829
**Gene: CCL19**							
**Feature**	**ϕi**	**Φi SD**	**Wald statistics**	**Φi 95% CI**	**Φi 95% CI**	**Predicted probability**	**p-value**
Intercept 1 (predicted state: ≥ 20 mm)	-0.8176	0.1256	42.350	-1.0638	-0.5713	0.207	<0.001
Intercept 2 (predicted states: 10 – 19 mm or ≥ 20 mm)	0.3926	0.1168	11.300	0.1637	0.6216	0.653	<0.001
CCL19 normal	-0.0019	0.0053	0.125	-0.0122	0.0085	-	0.724
CCL19 lesion	0.0250	0.0136	3.361	-0.0017	0.0518	-	0.067
CCL19 lesion/normal	-	-	-	-	-	-	0.388
CCL19 normal×CCL19 lesion×CCL19 lesion/normal	-	-	-	-	-	-	0.749
CCL19 normal×CCL19 lesion	-	-	-	-	-	-	0.494

SD, standard deviation; CI, confidence interval.

Table S4. Contingency table for chemokine protein expression in hyperplastic polyps from serrated pathway and adenomas (results of McNemar test)

Type category	Gene	Condition	Lesion (+ or ++)	Lesion (-)	p
Hyperplasia	CCL3	Margin (+ or ++)	1 [0.25]	0 [0.00]	0.2500
		Margin (-)	3 [0.75]	0 [0.00]	
	CCL4	Margin (+ or ++)	1 [0.25]	1 [0.25]	1.0000
		Margin (-)	0 [0.00]	2 [0.50]	
	CXCL2	Margin (+ or ++)	4 [1.00]	0 [0.00]	-
		Margin (-)	0 [0.00]	0 [0.00]	
	CCL19	Margin (+ or ++)	3 [1.00]	0 [0.00]	-
		Margin (-)	0 [0.00]	0 [0.00]	
Adenoma (all types)	CCL3	Margin (+ or ++)	23 [0.40]	5 [0.09]	0.0061
		Margin (-)	19 [0.33]	10 [0.18]	
	CCL4	Margin (+ or ++)	41 [0.71]	6 [0.10]	0.6072
		Margin (-)	9 [0.16]	2 [0.03]	
	CXCL2	Margin (+ or ++)	0 [0.00]	2 [0.03]	0.0225
		Margin (-)	11 [0.19]	45 [0.78]	
	CCL19	Margin (+ or ++)	51 [0.88]	0 [0.00]	0.0156
		Margin (-)	7 [0.12]	0 [0.00]	
